# PML-IRIS during Fingolimod Diagnosed after Natalizumab Discontinuation

**DOI:** 10.1155/2014/307872

**Published:** 2014-11-23

**Authors:** J. Killestein, A. Vennegoor, A. E. L. van Golde, R. L. J. H. Bourez, M. L. B. Wijlens, M. P. Wattjes

**Affiliations:** ^1^Department of Neurology, MS Center Amsterdam, VU University Medical Center, P.O. Box 7057, 1007 MB Amsterdam, The Netherlands; ^2^Department of Neurology, ZGT, Almelo, The Netherlands; ^3^Department of Radiology, ZGT, Almelo, The Netherlands; ^4^Department of Radiology, Nuclear Medicine & PET Research, MS Center Amsterdam, VU University Medical Center, Amsterdam, The Netherlands

## Abstract

*Background.* Natalizumab treatment is frequently discontinued and replaced by alternative medication in multiple sclerosis (MS) patients having a high risk of progressive multifocal leukoencephalopathy (PML). *Case Presentation.* We report a PML case that was missed on magnetic resonance imaging (MRI) at the time Natalizumab treatment was discontinued. The patient subsequently developed a PML-immune reconstitution inflammatory syndrome after the initiation of Fingolimod treatment, suggesting that immune reconstitution may occur even during Fingolimod induced lymphopenia. *Conclusion.* This report highlights the need for strict drug surveillance using MRI of Natalizumab-associated MS patients at the time of drug discontinuation and beyond. This is important with respect to pharmacovigilance purposes not only for Natalizumab, but also for alternative drugs used after Natalizumab discontinuation.

## 1. Introduction

Progressive multifocal leukoencephalopathy (PML) is a serious adverse event in multiple sclerosis (MS) patients treated with Natalizumab (Tysabri, Biogen Idec, MA, USA) [1]. Many patients who display anti-JCV antibodies decide to switch to Fingolimod (Gilenya, Novartis) or alternative drugs. There are unaddressed issues regarding the best possible regimen for switchers, including the right time point to start Fingolimod after Natalizumab discontinuation to avoid possible MS rebound. Recent studies suggest less prominent rebound disease breakthrough with shorter Natalizumab wash-out periods [2–4]. No PML cases related to Fingolimod monotherapy have been reported so far. Natalizumab-associated PML after drug discontinuation is a rather infrequent phenomenon, although the drug may remain detectable up to 200 days after cessation of therapy and induces shifts in circulating T-cell subpopulations that persist over several months [5–8].

Here we report a patient with an initially missed diagnosis of Natalizumab-associated PML at the time of drug discontinuation, who developed PML-immune reconstitution inflammatory syndrome (IRIS) after initiating Fingolimod treatment.

## 2. Case Report 

**Figure 1:**
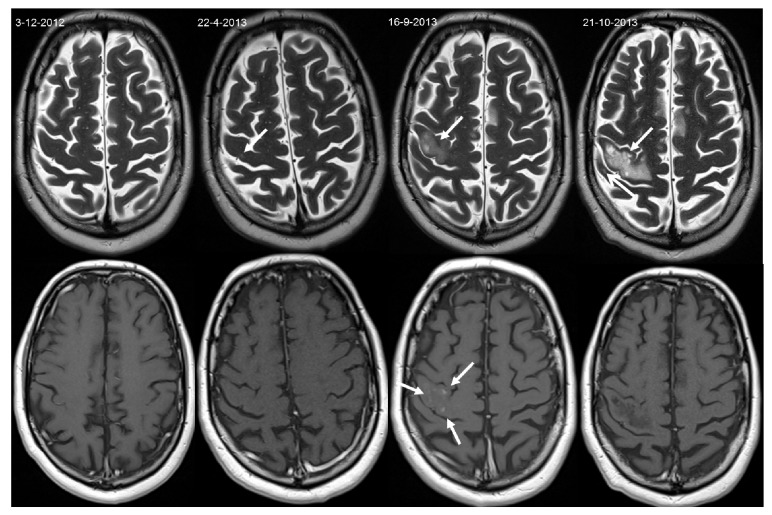
Axial T2-weighted (top row) and contrast-enhanced T1-weighted images (bottom row) obtained during Natalizumab treatment (3 December 2012), at the time of Natalizumab discontinuation (22 April 2013), at the time of PML-IRIS occurrence (16 September 2013), and after PML-IRIS treatment (21 October 2013). A small lesion suggestive of PML was already visible at the time of Natalizumab discontinuation (arrow). During Fingolimod treatment, this lesion progressed and changed to a PML-IRIS manifestation with slight mass effect and contrast enhancement (arrows) leading to posttreatment sequelae (arrow).

A 52-year-old relapsing-remitting MS patient switched to Natalizumab treatment in 2011, due to a breakthrough of MS disease activity while being treated with Glatiramer (Copaxone, Teva). Due to a positive STRATIFY JCV antibody test (Unilabs) in the spring of 2013, Natalizumab was discontinued and Fingolimod initiated. At that time (22-04-2013), brain MRI showed a new, small lesion in the cortical grey matter of the right precentral gyrus, which, according to recently published PML imaging characteristics [9, 10], can be interpreted retrospectively as PML onset (see Figure 1). After 3 months of wash-out, Fingolimod (0.5 mg) was initiated on 31 July 2013. By early September 2013, the patient presented with partial epileptic seizures of the left arm, which responded well to carbamazepine. However, in the following days after the seizures ceased, he developed an increase in fatigue and difficulties in fine hand movements, as well as mild weakness of his left arm, initially interpreted as MS exacerbation. Subsequently, he received IV methylprednisolone (1000 mg) for three days from 8 September 2013. MRI was repeated on 10 September 2013 and showed lesions highly suggestive of PML. At that stage, the patient fulfilled the diagnostic criteria of “possible PML” according to the AAN diagnostic criteria [11]. Fingolimod was discontinued. On 16 September 2013, brain MRI showed evolution and an enhancement pattern suggestive of PML-IRIS (Figure 1). IV methylprednisolone (1000 mg) for three days was repeated weekly until the 19th of October 2013.

Cerebrospinal fluid multiplex quantitative polymerase chain reaction performed at the US National Institute of Health Laboratory of Molecular Medicine and Neuroscience on 11 September 2013 did not reveal JCV DNA copies. However, radiological characteristics and the disease course make alternative diagnoses very unlikely.

Lymphocyte counts were measured before, during, and after Fingolimod treatment (see Table 1), suggesting that immune reconstitution may occur even during Fingolimod induced lymphopenia.

## 3. Discussion

No cases of PML have been described in patients using Fingolimod monotherapy most likely because it clearly differs from Natalizumab in pharmacodynamic immune mechanisms of action and subsequently its safety profile. This case study documenting the development of PML-IRIS during Fingolimod treatment after Natalizumab discontinuation backs up our conviction that a suspicion of a possible Fingolimod-associated PML in this setting has to be analysed very carefully. Current Natalizumab surveillance programs use MRI as a sensitive screening tool for PML detection preferably in the presymptomatic/asymptomatic stage, which is associated with a better prognosis [9, 10]. Our case is another argument that MRI surveillance should not stop at the time of Natalizumab discontinuation. Asymptomatic Natalizumab-associated PML can present with small lesions on MRI and may show a rather mild progression, staying asymptomatic for several months [12]. A recently published case series of 17 patients developing PML after Natalizumab discontinuation showed that this phenomenon is highly clinically relevant [7]. Interestingly, the majority of these patients developed PML within the first few months after drug discontinuation, suggesting that PML might already have occurred at the time of drug discontinuation or shortly thereafter [8]. In addition, small PML lesions can be missed, like in our case, or misinterpreted as a new MS lesion, supporting our opinion that Natalizumab surveillance, particularly the MRI assessment, should be committed to experienced centres. The need for professional drug surveillance in patients switching from one therapy to another is also relevant in terms of pharmacovigilance of drugs used after Natalizumab discontinuation. As the drug remains detectable in the blood up to 200 days after cessation of Natalizumab [5] and early detection of asymptomatic PML has a much better prognosis than symptomatic PML [9], we believe that MRI surveillance of high risk patients in terms of Natalizumab-associated PML should be performed every three months and continued at least 6 months after Natalizumab discontinuation.

Our case may also imply that the PML-IRIS phenomenon seems to start independently of the usage of Fingolimod, suggesting that lymphocytic cellular host-defence against JCV reactivation might not be (totally) impaired during Fingolimod treatment [13]; Fingolimod reduced CSF CD4+ T cells and B cells. However, the decrease was less pronounced than in the peripheral blood. In addition, the proportion of CSF CD8+ T cells, NK cells, and monocytes even increased compared to treatment-naive patients [13], supporting the use of Fingolimod after Natalizumab-associated PML in active MS.

Rebound disease activity after cessation of Natalizumab is well recognized and breakthroughs have been reported even in patients who switched to Fingolimod [2, 14]. Our observation confirms, however, that clinical deterioration after Natalizumab discontinuation should not automatically lead to the conclusion that we are dealing with a rebound of MS activity. The risk of further evolvement of Natalizumab-associated PML after stopping the drug is not imaginary. Since both situations can be clinically and radiologically difficult to differentiate, this is another strong argument to leave this challenging and potentially life threatening diagnostic issue in the hands of experienced physicians.

## Figures and Tables

**Table 1:** Lymphocyte counts before, during, and after Fingolimod treatment.

06-06-2013	3.2
17-08-2013	1.4
17-09-2013	0.9
24-09-2013	0.8
01-10-2013	0.8
04-10-2013	0.6
08-10-2013	2.8

Lymphocyte counts 2 × 10^9^/L (normal range 0.6–2.9). The final Fingolimod dose was administered on 11 September 2013. Increase in lymphocyte counts long after IRIS phase started (16 September 2013), suggesting that immune reconstitution occurs even in low lymphocyte state under Fingolimod.
